# Rapid and real-time identification of fungi up to species level with long amplicon nanopore sequencing from clinical samples

**DOI:** 10.1093/biomethods/bpaa026

**Published:** 2020-12-23

**Authors:** Sara D’Andreano, Anna Cuscó, Olga Francino

**Affiliations:** 1 SVGM, Servei Veterinari de Genètica Molecular, Universitat Autònoma de Barcelona, 08193, Bellaterra, Barcelona, Spain; 2 Vetgenomics, Edifici EUREKA, Parc de Recerca de la UAB, Campus UAB, 08193, Bellaterra, Barcelona, Spain

**Keywords:** fungi, nanopore, ribosomal operon, *Malassezia*, otitis, dog

## Abstract

The availability of long-read technologies, like Oxford Nanopore Technologies, provides the opportunity to sequence longer fragments of the fungal ribosomal operon, up to 6 Kb (18S-ITS1-5.8S-ITS2-28S) and to improve the taxonomy assignment of the communities up to species level and in real-time. We assess the applicability for taxonomic assignment of amplicons targeting a 3.5 Kb region (V3 18S-ITS1-5.8S-ITS2-28S D2) and a 6 Kb region (V1 18S-ITS1-5.8S-ITS2-28S D12) with the What’s in my pot (WIMP) classifier. We used the ZymoBIOMICS^TM^ mock community and different microbiological fungal cultures as positive controls. Long amplicon sequencing correctly identified *Saccharomyces cerevisiae* and *Cryptococcus neoformans* from the mock community and *Malassezia pachydermatis*, *Microsporum canis* and *Aspergillus fumigatus* from the microbiological cultures. Besides, we identified *Rhodotorula graminis* in a culture mislabelled as *Candida* spp. We applied the same approach to external otitis in dogs. *Malassezia* was the dominant fungal genus in dogs’ ear skin, whereas *Ma. pachydermatis* was the main species in the healthy sample. Conversely, we identified a higher representation of *Ma. globosa* and *Ma. sympodialis* in otitis affected samples. We demonstrate the suitability of long ribosomal amplicons to characterize the fungal community of complex samples, either healthy or with clinical signs of infection.

## Introduction

Fungi are a vast kingdom of organisms with a range between 1.5 and 6 million species [7], but only a modest part, around 140.000 species, is phenotypical and genetically described [8, 27]. Usually, fungi have been identified by morphology on pure cultures in agar medium. The main problem is that many species are difficult to isolate and culture, and even to classify [1, 24].

Both clinical approaches and research applications need taxonomic classification to assign taxa to their functional traits [6, 17]. On the contrary, problems of unknown branches of fungal phylogenies still occur, due to considerable gaps in genetic knowledge and to old species description [22].

Sequence-based methods allow to classify the fungi kingdom better. Still, the choice of the methodology used to study the mycobiome, or even the intrinsic characteristics of a specific fungus, can impact the data generated and the results reached [24]. Public online databases for fungal identification are noteworthy but still limited due to new updates for the best nomenclature and identification of fungi species [16]. A large number of online fungal databases are available for mycology research, and they are growing thanks to the dedication of the experts’ team. Taxonomic revisions are still ongoing and the main databases for fungi classification are Species Fungorum (www.speciesfungorum.org), MycoBank (www.mycobank.org), UNITE [13] and International Nucleotide Sequence Database Consortium (https://www.ncbi.nlm.nih.gov/taxonomy).

**Figure 1:**
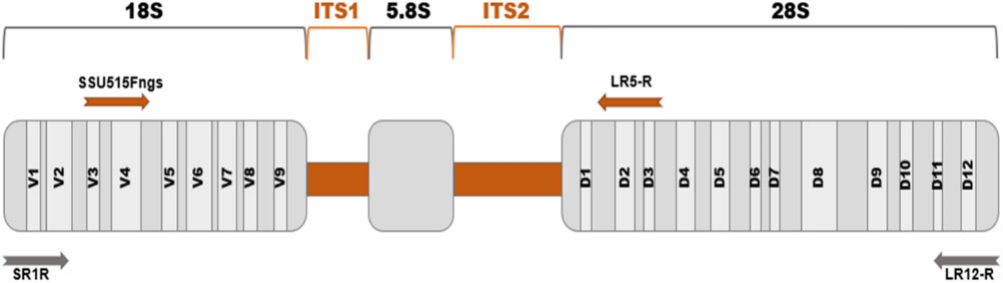
Fungal ribosomal operon: two hypervariable internal transcribed spacers regions (ITS1 and ITS2, marked in orange) and three conserved ones (18S, 5.8S and 28S rRNA, marked in grey) that contain variables domains, nine for the 18S and twelve for the 28S rRNA genes. Primers set used for the amplification of the ITS region (3.5 Kb) are shown in orange in the upper part of the operon, and the ones for amplification of the full operon (6 Kb) in grey in the lower part [20, 25].

One of the preferred markers for taxonomy assignment is the fungal ribosomal operon, which is almost 6.000 bp length. It contains three conserved units, the 18S rRNA gene (small subunit, SSU), 5.8S rRNA gene and 28S rRNA gene (large subunit, LSU), and two hypervariable units as internal transcribed spacers regions (ITS1 and ITS2). The ITS units flank the 5.8S RNA gene, and better represent the high variability among taxonomic levels of fungi; showing a superior species discrimination and PCR success rates [10]. The variable domains located at the conserved 18S (V1-V9) and 28S rRNA genes (D1-D12) (Fig. 1) are also worth considering to refine the taxonomy assignment. It is essential to recognize that the D1-D2 from the 28S rRNA gene domains are the ones that perform a higher level of taxonomic assignment for fungi [20].

Primer sets to amplify the fungal operon regions have been described in different manuscripts, starting from 1990 until 2018 [9, 12, 21, 22, 25, 26]. These sets of primers target the appropriate operon fragments to proceed with either short fragments, by massively parallel sequencing (or second generation sequencing) as Ion Torrent or Illumina, or longer fragments with single-molecule sequencing (or third generation sequencing) as PacBIO or Oxford Nanopore Technologies.

Taxonomy with short reads is focused on ITS1 and ITS2 regions, considered as the official barcoding markers for species-level identification in fungi, due to their easy amplification, conserved primers sites, widespread use [19] and available databases, such as UNITE or MycoBank. Usually, the ITS1 and ITS2 regions provide the taxonomy resolution within-genus and within-species level, but debates on which one provides the best taxonomic skill are still under discussion [2]. Alternative markers located in the small and large subunits of the rRNA genes can address the phylogenetic diversity, depending on the fungi species [2, 20]. Depending on the fungus species, different regions of the operon can be considered for taxonomy assignment: the SSU and the LSU are used when taxonomy is investigated up to family level, while lower taxonomy level analysis requires the ITS regions. When primers sets include the D1-D2 regions of LSU subunit, fragments obtained from the amplification can be assigned up to the species level [17].

Here, we aim to investigate the applicability for taxonomic assignment of the long-amplicon PCR approach to detect and identify the fungal microbiota present on complex microenvironments at the species level. We use microbiological fungal cultures characterized phenotypically as positive controls. We then apply the same protocol to clinical samples of canine otitis as a complex microenvironment.

## Materials and methods

### Samples and DNA extraction

LETI laboratories (LETI Animal Health) kindly provided a total of eight microbiological fungal cultures grown at 28°C in Petri dishes in Dermatophyte Test Medium (DTM agar) and Sabouraud Chloramphenicol agar. Four of the cultures had been classified up to the genus level (*Alternaria* spp., *Aspergillus* spp., *Candida* spp. and *Malassezia* spp.) and four other ones up to the species level: three of *Malassezia pachydermatis* and one of *Microsporum canis.* Also, fungal DNA of the ZymoBIOMICS^TM^ mock community (Zymo Research, Irvine, CA) containing *Saccharomyces cerevisiae* and *Cryptococcus neoformans* was included in the study as a positive control. The DNA from all fungal samples was extracted by ZymoBIOMICS^TM^ Miniprep kit following the manufacturer’s instructions.

Four canine otitis samples were analysed as complex microbial microenvironments. Two of them were collected from a Petri dish, divided into two halves parts, to culture fungi from both ears of a dog, one of the ears was healthy and the other one showed clinical signs for external otitis (S02_healthy; S03_affected). The DNA was extracted by using ZymoBIOMICS^TM^ Miniprep kit, as for the cultures. The other two otitis samples (S01_affected; S04_affected) were collected by swabbing the inner pinna of the ear of two dogs using Sterile Catch-All™ Sample Collection Swabs (Epicentre Biotechnologies). DNA was extracted with QIAGEN-DNeasy PowerSoil Kit (Hilden, Germany). DNA quality control was checked by Nanodrop and Qubit™ Fluorometer (Life Technologies, Carlsbad, CA).

### MinION sequencing

Two sets of primers were chosen (Table 1; Fig. 1): the first set targeting the ribosomal operon from V3 region of 18S RNA gene to D3 region of 28S RNA gene (≈3.500 bp), and the second one targeting the complete ribosomal operon from V1 region of 18S RNA gene to D12 region of 28S RNA gene (≈6000 bp). The primers, both forward and reverse, included the Nanopore Universal Tags (Oxford Nanopore Technologies Ltd, UK).

**Table 1:** Primers targeting the full ITS region (3.5 Kb) and the whole fungal operon (6 Kb)

Name	Sequence (5′-3′)	Target	Amplicon	Reference
SSU515Fngs-F	**TTTCTGTTGGTGCTGATATTGC**GCCAGCAACCGCGGTAA	18S-V3	3.5 Kb	Tedersoo et al. [20]
LR5-R	**ACTTGCCTGTCGCTCTATCTTC**TCCTGAGGGAAACTTCG	28S-D3	3.5 Kb	Tedersoo et al. [20]
SR1R-Fw	**TTTCTGTTGGTGCTGATATTGC**TACCTGGTTGATQCTGCCAGT	18S-V1	6 Kb	Vilgalys Lab [25]
LR12-R	**ACTTGCCTGTCGCTCTATCTTC**GACTTAGAGGCGTTCAG	28S-D12	6 Kb	Vilgalys Lab [25]

The Nanopore Universal Tag in bold type.

Two PCRs were performed: the first for the amplification of the target and the second one to add the specific barcode to each sample. DNA initial concentration was of 5 ng DNA per sample, in 50 μl of PCR final volume: 15 μl of DNA plus 35 μl of PCR mix, which contained 10 μl of Phusion^®^ High Fidelity Buffer (5×), 5 μl of dNTPs (2 mM), 0.5 μM of primer forward and reverse, and 0.02 U/μl of Phusion^®^ Hot Start II Taq Polymerase (Thermo Fisher Scientific GmbH, Dreiech, Germany). PCR profile included an initial denaturation of 30 s at 98°C, followed by 25 cycles of 10 s at 98°C, 30 s at 62°C, 80 s at 72°C and a final extension of 10 min at 72°C. Amplicons obtained were purified with Agencourt AMPure XP beads (Beckman Coulter™ A63880, Thermo Fisher Scientific GmbH, Dreiech, Germany), at 0.4× ratio for the fungal amplicon; then, they were quantified by Qubit™ fluorometer (Life Technologies, Carlsbad, CA).

Following the PCR Barcoding kit protocol (SQK-PBK004; Oxford Nanopore Technologies Ltd, UK), 0.5 nM per each sample was required for the second PCR to add barcodes of PCR barcoding kit (EXP-PBC001). The final volume of second PCR is 100 μl, containing 20 μl of DNA template from the previous PCR at 0.5 nM, 2 μl of specific barcode and 78 μl of mixture that include 20 μl of 5× Phusion^®^ High Fidelity Buffer, 10 μl of dNTPs (2 mM) and 2 U/μl of Phusion^®^ Hot Start II Taq Polymerase. PCR profile included an initial denaturation of 30 s at 98°C, 15 cycles of 10 s at 98°C, 30 s at 62°C, 80 s at 72°C and final step of 10 min at 72°C. The amplicon obtained were purified again with Agencourt AMPure XP beads, at 0.4× ratio and quantified by Qubit™ fluorometer (Life Technologies, Carlsbad, CA).

We proceeded then to the Library preparation with the Ligation Sequencing kit (SQK-LSK109, Oxford Nanopore Technologies Ltd, UK). Barcoded samples (1.5 μg) were pooled in 47 μl of nuclease-free water and the library was prepared following the manufacturer conditions.

With a final step of Agencourt AMPure XP beads 0.4×, the DNA library was cleaned and ready to be loaded into the flow cell. We used two SpotON Flow Cells (FLO-MIN106; Oxford Nanopore Technologies Ltd, UK) for three MinION runs, primed with a mixture of sequencing buffer and Flush buffer according to the manufacturer’s instructions. A quality control of sequencing pores was done before each run. Libraries were mixed with Sequencing Buffer and Loading Beads in a final volume of 75 μl. The final mix was added, by dropping, in the SpotON sample port.

Sequencing runs were performed using the MinKNOWN 2.2 v18.07.2 and the MinKNOWN v18.12.9 (Oxford Nanopore Technologies Ltd, UK). Nanopore sequencing from Oxford Nanopore Technologies includes real-time analysis with the EPI2ME platform [*What’s in my pot*, (WIMP)], allowing the identification of the fungal species few minutes after the run started.

### Data analysis

For further in-depth analyses, the fast5 files with the sequencing reads were basecalled and demultiplexed by Albacore v2.3.3 for the 3.5 Kb amplicon or guppy 2.3.5 for the 6 Kb amplicon. Barcodes and adapters were removed by using Porechop (https://github.com/rrwick/Porechop). Taxonomy was assigned with the cloud-based analysis WIMP application from the EPI2ME platform (Oxford Nanopore Technologies Ltd, UK), which is based on Centrifuge (https://ccb.jhu.edu/software/centrifuge/manual.shtml).

The fastq files output of each fungal amplicon with the length of 3.5 Kb and 6 Kb are loaded on Zenodo (http://doi.org/10.5281/zenodo.3662300) and ENA (PRJEB41658).

## Results

We aim to develop a long-amplicon PCR approach to detect and identify fungal microbiota present on complex microenvironments, and to apply it to clinical samples (canine otitis). As positive controls, we chose microbiological fungal cultures and fungal strains from a mock community. Some of the cultures were previously classified by classical microbiology up to the genus level as *Alternaria* spp., *Aspergillus* spp., *Candida* spp. and *Malassezia* spp*.* Others were classified up to the species level as *Ma. pachydermatis* and *Mi. canis* at LETI laboratories (LETI Animal Health). The ZymoBIOMICS^TM^ mock community fungal strains are *S. cerevisiae* and *C. neoformans.*

### Identification of microbiological cultures and mock community with long amplicons

**Figure 2:**
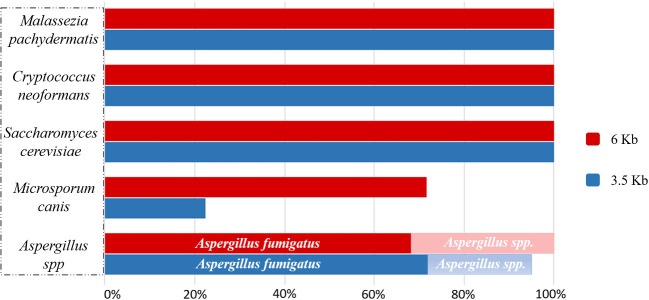
ZymoBIOMICS^TM^ mock community (*S. cerevisiae* and *C. neoformans*) and microbiological cultures of fungi after taxonomical classification of the 3.5 Kb and 6 Kb ribosomal amplicons.

All samples were amplified for both amplicon sizes, 3.5 Kb and 6 Kb. In the 3.5 Kb amplicons, we included those domains that better help in the taxonomic classification of fungi, as shown in Fig. 2.

Both amplicons correctly detected and identified the ZymoBIOMICS^TM^ mock community fungal strains (*S. cerevisiae*, *C. neoformans)*, and *Ma. pachydermatis* and *Mi. canis* from microbiological cultures. Looking in detail, *S. cerevisiae*, *C. neoformans* and *Ma. pachydermatis* were detected up to 100% by both 3.5 Kb and 6 Kb long fragments, while 6 Kb amplicon better detected *Mi. canis.*

**Figure 3:**
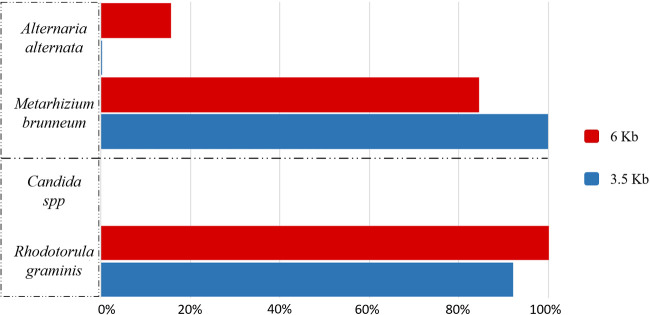
Fungal microbiological cultures showed unexpected results in the taxonomical classification after sequencing. Few reads from the *Alternaria* culture belonged to *Alternaria* spp., and it was classified at species level as *A. alternata*, but the most abundant fungus found was *Me. brunneum*. No reads from the *Candida* culture were classified as *Candida* spp. because of the presence of *R. graminis*.

Both fragments identified *Aspergillus* genus as the main one found in the culture, but looking at the species level, *Aspergillus fumigatus* was the most abundant. *Alternaria* spp. and *Candida* spp. showed different results from what we expected (Fig. 3).

For the *Alternaria* culture, both amplicons size detected *Metarhizium brunneum* as the main fungus found in these cultures. Conversely, *Alternaria alternata* was the species found in really low relative abundance (0.2% for 3.5 Kb and 15.6% for 6 Kb). No similarities between these fungi were found: they belong to different order, Pleosporales and Hypocreales*. Alternaria* is ubiquitous filamentous fungi, which are found in soil air and human/animal skin [15]; *Metarhizium* is commonly found as a parasite of insects and symbiont of plants [18, 23].

Looking at the nomenclature of this fungus, *Me. brunneum* belonged to *Me. anisopliae* strain [23, 29], but no correlation with *Al. alternata* was found.

For the *Candida* culture, the colonies of this fungus in Petri dish were red/orange. Sequences revealed the presence of *Rhodotorula graminis*, and only a few reads were classified as *Candida* spp*. Rhodotorula* is a carotenoid biosynthetic yeast, part of the Basidiomycota phylum, easily identifiable by distinctive yellow, orange or red colonies [28]. This yeast produces three major carotenoids: b-carotene, torulene and torularhodin, and is commonly associated with plants (http://www.antimicrobe.org/f16.asp#t1). In this case, we could confirm the presence of two yeast species in the microbiological culture.

#### Canine Otitis

Conscious that no differences were found in *Ma. pachydermatis* analysis using both fragments sizes (Fig. 2), we sequenced microbiological cultures of *Ma. pachydermatis* as positive controls and four complex samples with 3.5 Kb amplicon size. We run WIMP for fungal communities’ detection: the positive controls were identified as *Ma. pachydermatis*, while the complex otitis samples showed other *Malassezia* species (Table 2). The reads corresponding to *Malassezia* out of the total reads were 15 726 out of 15 940 for S02; 22 666 out of 23 067 for S03; 7188 out of 7842 for S04 and 15 243 out of 17 150 for S01.

**Table 2:** Relative abundance of *Malassezia* species found in pure *Ma. pachydermatis* culture, and in four complex samples belonged to three different dogs affected by otitis

Sample	*Malassezia pachydermatis* (%)	*Malassezia globosa* (%)	*Malassezia sympodialis* (%)	*Malassezia* spp. (%)	Others (%)
M01	98.9	0.2	0.2	0.2	0.5
M02	99.0	0.1	0.2	0.2	0.5
M03	98.9	0.2	0.3	0.2	0.4
S02_healthy	98.2	0.1	0.2	0.2	1.3
S03_affected	95.1	1.1	1.7	0.4	1.7
S04_affected	78.8	4.0	5.0	3.9	8.3
S01_affected	73.7	4.7	5.1	5.3	11.2

Samples S02 and S03 belong to the same dog, while S04 and S01 belong to two different dogs.

Two of the samples correspond to the same dog, one from a healthy ear (S02) and the other one (S03) with clinical signs compatible with otitis externa, and *Ma. pachydermatis* is the main fungal species detected in both ears. The other two samples (S01 and S04) came from the ear with otitis externa of two dogs. In that case, other *Malassezia* species were detected together with *Ma. pachydermatis*, such as *Ma. globosa* and *Ma. sympodialis* (Table 2).

## Discussion

Our first approach with Oxford Nanopore Technologies sequencing was aimed to understand if long amplicons are suitable markers to analyse the mycobiome in dog skin, and which size could be the best in the analysis of mycobiome. The microbiological cultures were essential for the study as positive controls because their genome sequences were used to validate the correct detection of fungi in WIMP.

Primers used to amplify the fungal ribosomal operon domains should be chosen depending on the fungus, but no standard markers are defined yet. The longest amplicons should be considered to describe the communities at lower taxonomy classification [22, 27]. *Malassezia* spp., *S. cerevisiae*, *C. neoformans*, *Mi. canis* and *Aspergillus* spp. were correctly detected and identified from the microbiological cultures. However, the microbiological cultures corresponding to *Alternaria* spp. and *Candida* spp. were misidentified as per classical microbiology, and other fungi were detected. It is noteworthy that the samples plated came from dog skin, which is prone to environmental contamination, as has been previously described in skin microbiome of healthy dogs [4, 5].

Few of the reads from the *Alternaria* culture were classified as *A. alternata* with the 6 Kb amplicon, while most classified as *Me. brunneum*. Discovered in Spain and used as an herbicide against fly *Bactrocera oleae* [29], this fungus belongs to the same phylum of Ascomycota, but it differs at lower taxonomy levels. The *Candida* microbiological culture was misclassified, even when showing an orange colour, caused by *R. graminis*.

In this study, the proper positive controls were those from the ZymoBIOMICS^TM^ mock community (*S. cerevisiae* and *C. neoformans*). The microbiological cultures were grown from dogs’ skin samples and donated after morphological classification by microbiologists as potential positive controls. We checked them with the long amplicon approach to reach the species level (when and if possible). The *Candida* and *Alternaria* colonies were originally misclassified, confirming that morphology only is not enough for fungal taxonomic classification.

Finally, we investigate the possibility of reaching species level in complex samples from the skin of dogs affected by otitis, finding that *Malassezia* was the most abundant genus. The classification at the species level was performed to investigate possible changes between health status and diseased one.

*Malassezia pachydermatis* has been reported as the most abundant species in the ear canal of healthy dogs [11]. WIMP correctly identified all the *Malassezia* samples, and we were able to identify *Malassezia* at the species level from four complex canine otitis samples. Two of the samples corresponded to the same dog, one from a healthy ear (S02) and one with clinical signs (S03) that were compatible with otitis externa. *Malassezia pachydermatis* is the main fungal species detected (98% of the reads for the healthy ear – S02 – and 95% for the sample with clinical signs compatible with otitis externa – S03). The other two samples (S01 and S04) came from the ear with otitis externa of two other dogs. In those cases, other *Malassezia* species were detected together with *Ma. pachydermatis*, such as *Ma. globosa* and *Ma. sympodialis*.

The results agree with previous studies on *Malassezia* spp., describing it as a commensal microorganism in human and animal skin that may become pathogenic [3, 14].

It is worthy to note that WIMP taxonomic assignation is based on Centrifuge, which could misclassify some closely-related species due to the absence of complete reference genomes in the database used. However, *Malassezia* is a genus with reference genomes for each one of its species, and such a misclassification is not observed for the positive control or the two other samples. The procedure will benefit by using a specific database of fungal ribosomal operons for the molecular identification of fungi, such as UNITE (https://unite.ut.ee), as there are many more ribosomal operons sequenced than complete fungal genomes. However, UNITE targets only the ITS region as the formal fungal barcode. The long-read sequencing either from fungal isolates or from microbiome samples will improve the database, which could be used with WIMP for the taxonomic assignment of fungi.

This study is a first approach for the applicability of ribosomal long amplicons to identify some of the most common fungi in the skin of dogs. We evaluate the applicability of long reads to the fungal ribosomal operon as a whole, taking into account not only ITS1 and ITS2 as the most common markers for fungal taxonomy, but adding the variable regions of 18S and 28S rRNA genes in the 6 Kb amplicon. We used positive controls from the ZymoBIOMICS^TM^ mock community, and from microbiological cultures that cannot always be considered pure cultures. We demonstrate the suitability of this approach to characterize the fungal community of otitis samples, either healthy samples or samples with clinical signs of infection. It is out of the scope of this study the comparison with other non-ribosomal loci commonly used for taxonomic assignment when ITS is not able to resolve to species level. Further steps are needed to evaluate the ribosomal long amplicon as a potential universal barcode, without discarding the need to rely on non-ribosomal loci for sequence-based identification of some fungal taxa.

On the contrary, nanopore sequencing is a technology that evolves fast and both the library preparation and the bioinformatics tools improve every few months for providing better results in real time. The next steps will lead to simplify the library preparation with the Rapid Barcoding kit from ONT and the analysis of complex samples from different origins to detect the causal agent of the disease in a clinical metagenomics approach.

## Data Availability

The fastq files output of each fungal amplicon with the length of 3.5 Kb and 6 Kb are loaded on Zenodo (http://doi.org/10.5281/zenodo.3662300).

## Funding

This study was funded by a grant awarded by the Generalitat de Catalunya (Industrial Doctorate Program, grant 2015DI044) and by Vetgenomics.

*Conflict of interest*: AC and OF work for Vetgenomics, SL.
